# Extending long-range phasing and haplotype library imputation algorithms to large and heterogeneous datasets

**DOI:** 10.1186/s12711-020-00558-2

**Published:** 2020-07-08

**Authors:** Daniel Money, David Wilson, Janez Jenko, Andrew Whalen, Steve Thorn, Gregor Gorjanc, John M. Hickey

**Affiliations:** The Roslin Institute and Royal (Dick) School of Veterinary Studies, The University of Edinburgh, Easter Bush, Midlothian, Scotland, UK

## Abstract

**Background:**

We describe the latest improvements to the long-range phasing (LRP) and haplotype library imputation (HLI) algorithms for successful phasing of both datasets with one million individuals and datasets genotyped using different sets of single nucleotide polymorphisms (SNPs). Previous publicly available implementations of the LRP algorithm implemented in AlphaPhase could not phase large datasets due to the computational cost of defining surrogate parents by exhaustive all-against-all searches. Furthermore, the AlphaPhase implementations of LRP and HLI were not designed to deal with large amounts of missing data that are inherent when using multiple SNP arrays.

**Methods:**

We developed methods that avoid the need for all-against-all searches by performing LRP on subsets of individuals and then concatenating the results. We also extended LRP and HLI algorithms to enable the use of different sets of markers, including missing values, when determining surrogate parents and identifying haplotypes. We implemented and tested these extensions in an updated version of AlphaPhase, and compared its performance to the software package Eagle2.

**Results:**

A simulated dataset with one million individuals genotyped with the same 6711 SNPs for a single chromosome took less than a day to phase, compared to more than seven days for Eagle2. The percentage of correctly phased alleles at heterozygous loci was 90.2 and 99.9% for AlphaPhase and Eagle2, respectively. A larger dataset with one million individuals genotyped with 49,579 SNPs for a single chromosome took AlphaPhase 23 days to phase, with 89.9% of alleles at heterozygous loci phased correctly. The phasing accuracy was generally lower for datasets with different sets of markers than with one set of markers. For a simulated dataset with three sets of markers, 1.5% of alleles at heterozygous positions were phased incorrectly, compared to 0.4% with one set of markers.

**Conclusions:**

The improved LRP and HLI algorithms enable AlphaPhase to quickly and accurately phase very large and heterogeneous datasets. AlphaPhase is an order of magnitude faster than the other tested packages, although Eagle2 showed a higher level of phasing accuracy. The speed gain will make phasing achievable for very large genomic datasets in livestock, enabling more powerful breeding and genetics research and application.

## Background

Here, we describe the latest improvements to the long-range phasing (LRP) [1] and haplotype library imputation (HLI) algorithms, as implemented in the AlphaPhase software [2], to phase genotypes for hundreds of thousands of individuals that may have been genotyped on different platforms. Phasing genotypes is the process of inferring the parental origin of an individual’s alleles. This process resolves the inheritance of chromosome segments in a population and is, as such, a cornerstone technique in genetics. For example, it is useful for making genotype calls, imputing genotypes, detecting phenotype-genotype associations in the presence of effects such as allele-specific expression, and inferring recombination points and demographic history [3].

The size of genomic datasets has grown rapidly in recent years, with genotype data from single nucleotide polymorphism (SNP) arrays being collected on increasing numbers of individuals. In agriculture, this growth has been driven by the increased use of genomic selection [4–6], whereas in human genetics it has been driven by the increased power of genome-wide association studies [7–9] and of genomic prediction in human medicine [10]. Examples of such large datasets include the UK Biobank [11], which has recently released SNP genotype data on approximately half a million people [12], and the US Dairy Cattle and Irish Cattle Breeding Federation Databases, which each host genotypes on well over a million animals [6, 13, 14].

In many cases, these datasets have been collected over several years and have been genotyped using different SNP arrays [6, 14]. Methods such as SNPchiMp [15] have been developed to allow the manipulation of sets of genotypes from multiple SNP arrays, but the main aim of these methods is to ensure that the different sets are combined correctly rather than to perform analyses of the combined dataset.

Several methods for phasing genotype data have been developed based on probabilistic methods, such as those implemented in fastPHASE [16] and Beagle [17]. Others, such as AlphaPhase [2], findHap [18], and Fimpute [19], are based on heuristic methods. FindHap and FImpute have been shown to be capable of phasing very large datasets [18], potentially containing over one million individuals. Recent developments in probabilistic methods, e.g. SHAPEIT3 [20] and Beagle [17], have enabled these methods to also phase very large datasets [21].

Miar et al. [22] compared several phasing algorithms and found that FImpute was the fastest and most accurate algorithm for phasing large livestock populations, providing similar accuracy as Beagle [17] and SHAPEIT2 [23], while being significantly faster. In contrast Delaneau et al. [24], found that SHAPEIT2, in general, offered significantly better accuracy than Beagle on human datasets, while Loh et al. [25] showed that Eagle2 performs better than SHAPEIT2, both in terms of run time and accuracy. These observations suggest that there is not one single best phasing algorithm but that the best algorithm is dataset dependent.

AlphaPhase [2] is a heuristic method that combines LRP and HLI. LRP infers parental origin of alleles by finding surrogate parents of an individual, i.e. individuals who likely have the same haplotype as the individual. If a surrogate parent is homozygous for a SNP, then it can be used to phase that individual’s genotypes for that SNP. When a homozygous surrogate parent cannot be found, surrogate parents of the heterozygous surrogate parent can be used. This process is repeated, with increasingly remote surrogate parents, until the individual’s genotype can be phased. HLI infers the phase of a genotype by creating a library of haplotypes that are fully phased. Partially phased haplotypes can be fully phased by matching with library haplotypes.

Existing publicly available implementations of the LRP algorithm used in AlphaPhase cannot efficiently phase large datasets since finding surrogate parents among all the individuals in a population involves comparing every individual with every other individual. Both runtime and memory usage quickly become impractical with large datasets, as they scale with the square of the number of individuals. In addition, existing publicly available implementations of the LRP and HLI algorithms used in AlphaPhase cannot phase datasets that include different sets of markers, as they were not designed to cope with large amounts of missing data. Combining data from multiple SNP arrays can lead to large amounts of missing data.

In this paper, we introduce improvements that allow AlphaPhase to (a) perform LRP of large datasets and (b) perform both LRP and HLI with missing data. These improvements enabled us to quickly and accurately phase large heterogeneous simulated datasets.

## Methods

### Previous LRP and HLI algorithms

Throughout the Methods section, LRP and HLI refer to the LRP and HLI algorithms as they were implemented in AlphaPhase by Hickey et al. [2]. Both algorithms operate on genome regions that are referred to as cores. A core is a set of consecutive SNPs for which phasing is being attempted. For further details, refer to Hickey et al. [2].

LRP infers the phase of an individual by using other individuals that are known to share a haplotype with the individual, which are called “surrogate parents” (shortened here to “surrogates”) and are identified by finding no opposing homozygous markers at any position within a core. Then, these surrogates are partitioned into either paternal or maternal surrogates of the individual using pedigree information, if available. If pedigree information is not available, this assignment is arbitrary.

Homozygosity of a surrogate at a position enables phasing of the individual at that position. If no homozygous surrogate is found, then it may be possible to phase the individual by using surrogates of surrogates. This process can be continued to an arbitrary depth. In practice, the consensus of several homozygous surrogates is taken to allow for error in the process of determining surrogates or genotype data.

HLI infers phase by matching partially phased haplotypes to a library of known haplotypes. In the current algorithm in AlphaPhase, the initial library is constructed from the fully phased haplotypes that are found during LRP and by adding new haplotypes as they are discovered. New haplotypes are discovered when one haplotype of an individual is inferred, which, together with genotype information, determines the other haplotype of the individual. This process is iterated until no new haplotypes are found.

### Extending long-range phasing to large datasets

To address the problem of scaling LRP to large datasets, we modified the algorithm so that it is performed on subsets of individuals, after which the results from each subset are combined. By performing LRP on subsets, the runtime can be vastly reduced, because the search for surrogates has quadratic runtime scaling, and in the worst case involves an all-against-all search for surrogates, which is too time consuming when the dataset is very large, while splitting the data into subsets limits the search time. Subsets of individuals, without replacement, are chosen randomly so that every individual is in a subset. Then, the results from running LRP on these subsets are merged and HLI is run on the merged dataset. We refer to this as the sub-setting method.

Preliminary analyses showed that including individuals in multiple subsets did not offer a significant improvement in accuracy, but increased runtime significantly (data not shown). Including related individuals in subsets also decreased accuracy (data not shown), likely due to the use of a crude clustering method to find related individuals, in order to be able to run the algorithm without a pedigree.

### Extending long-range phasing and haplotype library imputation to heterogeneous datasets

The LRP algorithm was modified to enable the identification of surrogates in the presence of missing data. Missing data hinders identification of opposing homozygotes and thus has the potential to incorrectly identify surrogates. To alleviate this problem, we introduced an additional parameter that defines the required number of shared markers between two individuals before surrogacy is tested.

**Fig. 1:**
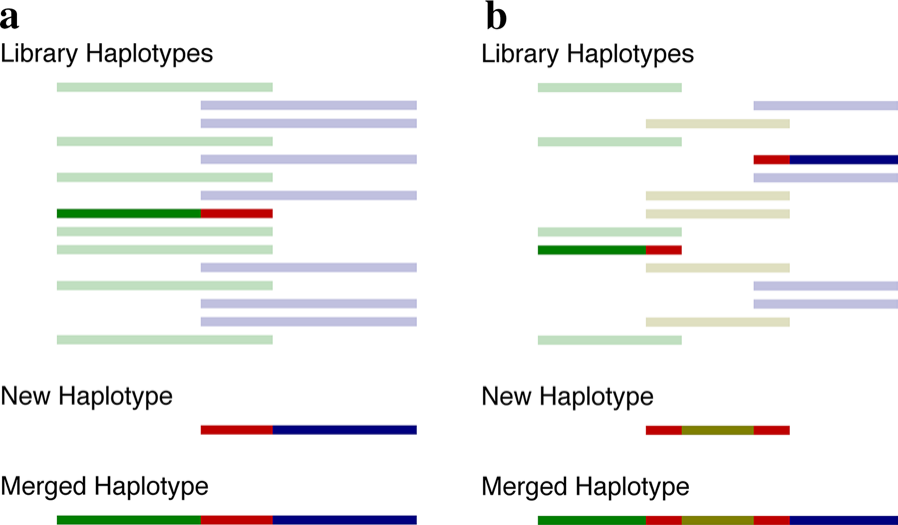
Modifications to the LRP and HLI algorithms to deal with library haplotypes with missing data. **a** In this example, we generated haplotypes using two different SNP arrays, as indicated by green and blue haplotypes. If the shared markers between two haplotypes are identical (shown in red), then the two haplotypes can be merged into one haplotype. To ensure the two haplotypes are the same haplotype, we set a minimum number of alleles that must be shared. Note that, in reality, both blue and green markers will be distributed along the length of the haplotype. **b** In this example, we generated haplotypes using three different SNP arrays. Finding the new purple haplotype allowed us to recognise that the green, purple, and blue haplotypes are actually the same haplotype

The HLI algorithm required more complex modifications to account for missing data. In a multiple SNP array setting, it is likely that most, or even all individuals, will have been genotyped with only one array, and thus will not have data for all markers that are present across arrays. The LRP algorithm cannot infer parental origin of alleles at missing markers. We developed HLI methods that allowed partially inferred haplotypes to be included in the haplotype library and to be used to infer other haplotypes. However, allowing for partially inferred haplotypes in the haplotype library severely complicates matching a new partially inferred haplotype to a library haplotype as it is necessary to ensure that the two haplotypes have enough markers with non-missing information to be confident that they are indeed the same haplotype. Thus, we added a parameter to the HLI algorithm that specifies the required number of shared alleles to match two haplotypes (Fig. 1a).

In some cases, the new haplotype matches more than one library haplotype, possibly because the library haplotypes are duplicates (Fig. 1b). In this situation, we merged the new haplotype and library haplotypes, replaced the incomplete library haplotypes with the merged haplotype, and updated individuals known to carry the original incomplete library haplotypes. If a new haplotype matched multiple library haplotypes and these matches could not be the same haplotype due to opposing homozygotes between the library haplotypes, then we added the new haplotype to the library.

### Speed and memory use optimization

Several changes were made to AlphaPhase to optimise it for speed and memory use. AlphaPhase was modified to store haplotypes and genotypes as bits and to use bit operations to operate on multiple SNPs at once, whenever possible. We also modified AlphaPhase to accept a haplotype library output from a previous AlphaPhase run. Therefore, it is now possible to run AlphaPhase on new individuals while including haplotype information from any individuals previously phased. AlphaPhase was also modified to further exploit high performance computing clusters. Previously, the only way to run AlphaPhase in parallel, was to run each chromosome separately. We have further improved AlphaPhase’s ability to run in parallel by adding options that allow each core to be run individually. These can then be concatenated back into results for the whole chromosome.

### Test datasets

**Fig. 2:**
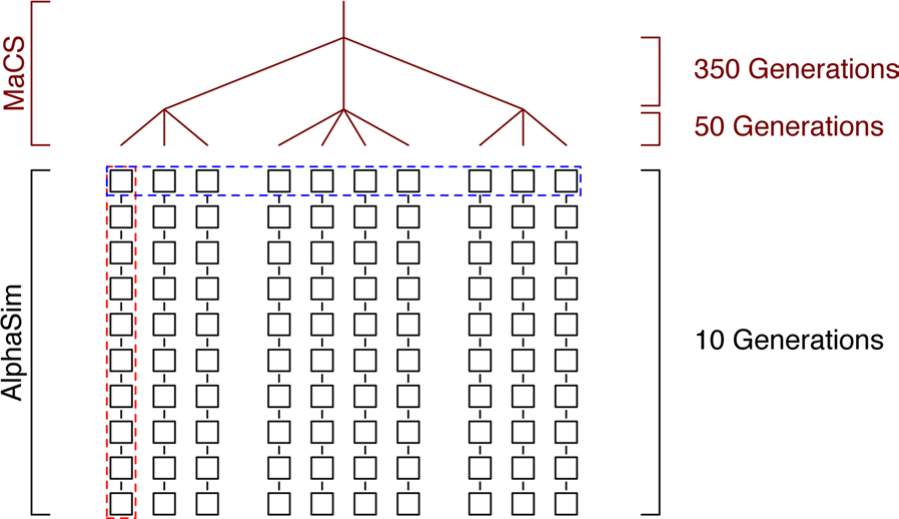
Structure of the simulated populations. MaCS was used to simulate a base population. This base population was generated from a single ‘breed’ that split into three breeds 400 generations ago. Fifty generations ago, each of these breeds split again into either three or four breeds to give ten breeds. Next, each of these ten breeds undergoes ten generations of selection using AlphaSim. The dotted blue line shows an example for the “per generation” scenario, while the dotted red line shows an example for the “per population” scenario

Performance of modified AlphaPhase algorithms was tested on large and heterogeneous datasets that were simulated using AlphaSim [26]. We followed the simulation scheme from [27], which we describe briefly and illustrate in Fig. 2. First, AlphaSim uses MaCS [28] to simulate base population haplotypes for a chromosome of 1 Morgan. We simulated a single ancestral breed, which split into three breeds 400 generations ago, each of these splitting again into either three or four breeds 50 generations ago, to give ten breeds of equal size. Two datasets consisting of the ten breeds were simulated, along with ten generations of selective breeding for each breed (Fig. 2). Selection was based on phenotype for a single trait that had 10,000 quantitative trait nucleotides with normally distributed effects. For the first dataset, we selected 25 sires and 500 dams to generate 1000 offspring. This resulted in a dataset of 100,000 animals (100 k dataset). The second dataset was created using 10,000 offspring for each breed and each generation to create a dataset of one million animals (one million dataset). For both datasets, one chromosome worth of SNP data was generated and SNPs with a minor allele frequency of at least 0.05 were chosen as possible candidates for inclusion on SNP arrays. The SNPchiMp software [15] was used to obtain information on the SNPs on different arrays and the overlap between arrays (as shown in Table 1). Chromosome 1 has 8771 unique SNPs across the bovine arrays. We selected this number of SNPs from the simulated candidate SNPs and then assigned SNPs to different arrays based on the pattern as reported by SNPchiMp for bovine arrays.

**Table 1:** Genotyping scenarios evaluated

Scenario	Description	Number of SNPs
Illumina 50 Kv2	Illumina 50 Kv2 (all)	6711
Illumina HD	Illumina HD (all)	49,579
Two-Illumina	Illumina 50 Kv1 and Illumina 50 Kv2 in a 1:1 ratio	6924
Two-mixed	Illumina 50 Kv2 and IDBv3 in a 1:1 ratio	7245
Three-MD	Illumina 50 Kv2, GSeekHD and IDBv3 in a 1:1:1 ratio	10,033
Mixed MD/HD	Illumina 50 Kv2 and Illumina HD in a 9:1 ratio	50,142

The assigned SNP arrays were then used to create scenarios (Table 1) in which individuals were genotyped with different arrays. For all scenarios, we simulated individuals as genotyped on different arrays by assigning them to arrays in proportions expected in real datasets (Table 1). Two scenarios consisted of genotypes from homogeneous arrays, where all individuals were genotyped with either the medium-density (MD) Bovine Illumina 50 Kv2 [29] or the high-density (HD) Illumina HD SNP [30] arrays. Five scenarios included heterogeneous arrays, where individuals were genotyped with a set of partially overlapping combinations of SNP arrays. Three of these scenarios were based on different MD chips. The two-Illumina scenario included two versions of the Illumina MD chip [29] (Illumina 50 Kv1 and Illumina 50 Kv2). The two-mixed scenario combined one Illumina chip (Illumina 50 Kv2) and one other chip (IDBv3 [31]). The three-MD scenario combined the Illumina 50 Kv2 chip with the IDBv3 chip and the GSeekHD chip [32]. The mixed MD/HD scenario combined a MD Illumina chip (Illumina 50 Kv2) with a HD Illumina chip (Illumina HD).

### Parameters used for AlphaPhase

AlphaPhase has several parameters that control phasing of alleles. Two of these were expected to have a significant effect on the performance of AlphaPhase: a parameter that controls core length (defined as the number of SNPs in each core) and a newly defined parameter that controls the size of phasing subsets to speedup phasing of a large dataset.

Core length can have a significant effect on phasing accuracy [2] and we tested different core lengths to find the best core length for both of the MD and HD scenarios. For the Illumina 50 Kv2 scenarios, we tested core lengths in the same range as those tested by Hickey et al. [2] for a similar size array: 50, 100, 200, 500, and 1000 SNPs. For the Illumina HD scenario, we tested core lengths of 500, 1000, 2000, 5000, and 10,000 SNPs because the Illumina HD array contains approximately ten times as many SNPs as the Illumina 50 Kv2 array.

We also tested different sizes of the phasing subsets, as this was expected to have an effect on phasing accuracy. Tested values were 500, 1000, 2000, 5000, and 10,000 individuals. For the Illumina 50 Kv2 scenario, we tested all combinations of core length and subset size. We only report subset size results for a fixed core length of 500 SNPs since the interaction between core length and subset size was minimal (data not shown). For the Illumina HD scenario, we set the core length to 5000 SNPs when subset size was tested.

For the heterogeneous array scenarios, we set the core length to 500 SNPs when the dataset consisted of only MD arrays, because this value gave good performance for the homogeneous MD array scenarios. Similarly, for datasets containing HD arrays, we set the core length to 5000 SNPs. We set subset size to 5000 individuals for all scenarios.

Fixed default values were used for several other parameters included in AlphaPhase. Specifically, we fixed the maximum number of surrogates used to 10 and allowed 10% of the marker genotypes to disagree between pairs of surrogates in order to be consistent with the original AlphaPhase study [2]. We also set the number of allele mismatches for clustering pairs of nearly identical library haplotypes to zero.

When phasing multiple arrays, we added two additional parameters to AlphaPhase. One parameter governs the minimum percentage of SNPs that need to be phased to add a haplotype to the library and we set this to 80%. The other parameter governs the minimum required number of matching alleles before two haplotypes can be identified as the same haplotype. If all SNPs are independent of each other, i.e., there is no linkage disequilibrium between SNPs, we expect the optimal value of this parameter to be the same regardless of SNP density. We tested different values of this parameter on both the Illlumina 50 Kv2 and Illumina HD datasets. Results (data not shown) suggested that presence of linkage disequilibrium did not have a significant effect on phasing accuracy for the SNP densities considered here and that requiring a match of 200 alleles between two haplotypes was appropriate for this parameter. If SNP arrays or sequence data with greater density are considered, the value of this parameter may need to be revised.

### Performance testing

To test the performance of the modifications of the LRP and HLI algorithms on large datasets, we used the data from the homogeneous array scenarios for both the 100 k and one million datasets. To test a scenario in which parents are known and for which genotype information is available, we evaluated phasing accuracy within each of the 10 breeds individually using data from all generations. Similarly, to test a scenario in which no parentage information is available, we evaluated phasing accuracy for each of the ten generations individually (Fig. 2). We report average results across either all ten breeds or across all ten generations.

To test the speed and memory usage of AlphaPhase on large datasets, we tested multiple combinations of numbers of generation and populations from both the 100 k and the one million datasets using homogeneous array scenarios. To test the performance of the modifications of the LRP and HLI algorithms on heterogeneous datasets we used the data from the heterogeneous array scenarios on the 100 k dataset.

To compare the performance of AlphaPhase to other phasing software, we compared it to Eagle2 [25] and Shapeit2 [23]. We found that Eagle2 outperformed Shapeit2 (results not shown), thus we report results for Eagle2 only. We ran Eagle2 using default parameters and assumed that a haplotype reference library was not externally available and had to be generated from the data. We did not filter out SNPs or individuals with a high rate of missingness. For the size test cases, we only ran Eagle2 on the 100 k Illumina 50 Kv2 dataset because of excessive Eagle2 run times.

We report three phasing statistics: percentage of correctly phased alleles, percentage of unphased alleles, and percentage of incorrectly phased alleles. Due to the presence of unphased alleles, the sum of the percentage of correctly and incorrectly phased alleles will not always sum to one hundred percent. Unless explicitly stated, we report these statistics for heterozygous loci only. We also report on memory usage and runtimes. Runs were performed on computers with an Intel Xeon Processor E5-2630 v3 (2.4 GHz) and between 64 and 1024 GB of RAM. In all cases, we report the total AlphaPhase runtime, and maximum memory usage, across all the individual core runs. We ran Eagle2 using a single thread in order to make runtimes comparable.

## Results

### Long range phasing and haplotype library imputation of large datasets

#### Core length

**Fig. 3:**
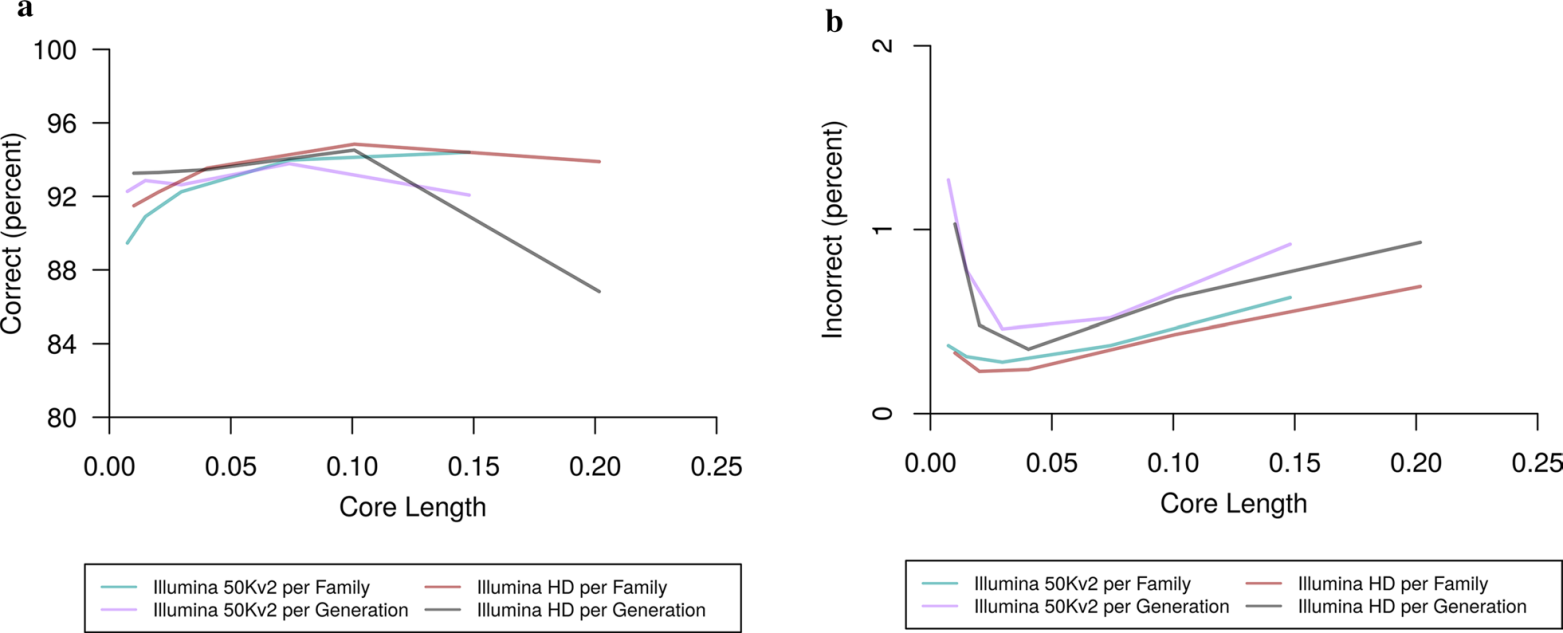
Phasing accuracy for a range of core lengths. **a** Percentage of correctly phased alleles at heterozygous loci. **b** Percentage of incorrectly phased alleles at heterozygous loci. Core lengths are given as a proportion of the total chromosome length

To determine the accuracy of the new sub-setting method for LRP, we first determined the optimal core length for each of the Illumina 50 Kv2 and Illumina HD scenarios. Figure 3 and Additional file 1: Tables S1 and S2 show the accuracy for the Illumina 50 Kv2 scenario for a variety of core lengths. Figure 3a shows the percentage of correctly phased heterozygous loci for the Illumina 50 Kv2 array per population scenario. The percentage of correctly phased alleles increased as the core length increased, although the difference in accuracy between a core length of 500 (94.0%) and 1000 SNPs (94.4%) was small. For the per generation scenario, the percentage of correctly phased alleles peaked at a core length of 500 SNPs (93.8%) before dropping significantly for a core length of 1000 SNPs (92.1%). The pattern for the number of incorrectly phased alleles for the Illumina 50 Kv2 scenario (Fig. 3b) was less clear, although there was a significant increase in the number of incorrectly phased alleles for a core length of 1000 SNPs. Using a core length of 500 SNPs, the percentage of alleles incorrectly phased was 0.4% for the per population scenario and 0.5% for the per generation scenarios, respectively).

Figure 3 and Additional file 1: Tables S3 and S4 show that for the Illumina HD scenario, the percentage of correctly phased alleles at heterozygous loci peaked at a core length of 5000 SNPs (per generation and per population). For this scenario, the number of incorrectly phased alleles was minimised at a core length of 1000 (0.2% per population) or 2000 SNPs (0.4% per generation), a shorter core length than that, which maximised the number of correctly phased markers. Using a core length of 5000 SNPs 94.8% (per population) and 94.5% (per generation) of alleles were phased correctly, while 0.4% (per population) or 0.6% (per generation) were phased incorrectly.

For all scenarios, runtime was found to be inversely proportional to core length (see Additional file 1: Tables S1–S4). We chose to study core lengths of 500 SNPs (for MD scenarios) and 5000 SNPs (for HD scenarios) as a reasonable trade-off between accuracy and runtime. For these core lengths, runtime was under 2 h for both arrays and for both the per generation and per population scenarios. Memory usage was 2.5 GB for the Illumina 50 Kv2 array and 4.0 GB for the Illumina HD array (Additional file 1: Tables S1–S4).

#### Subset size

**Fig. 4:**
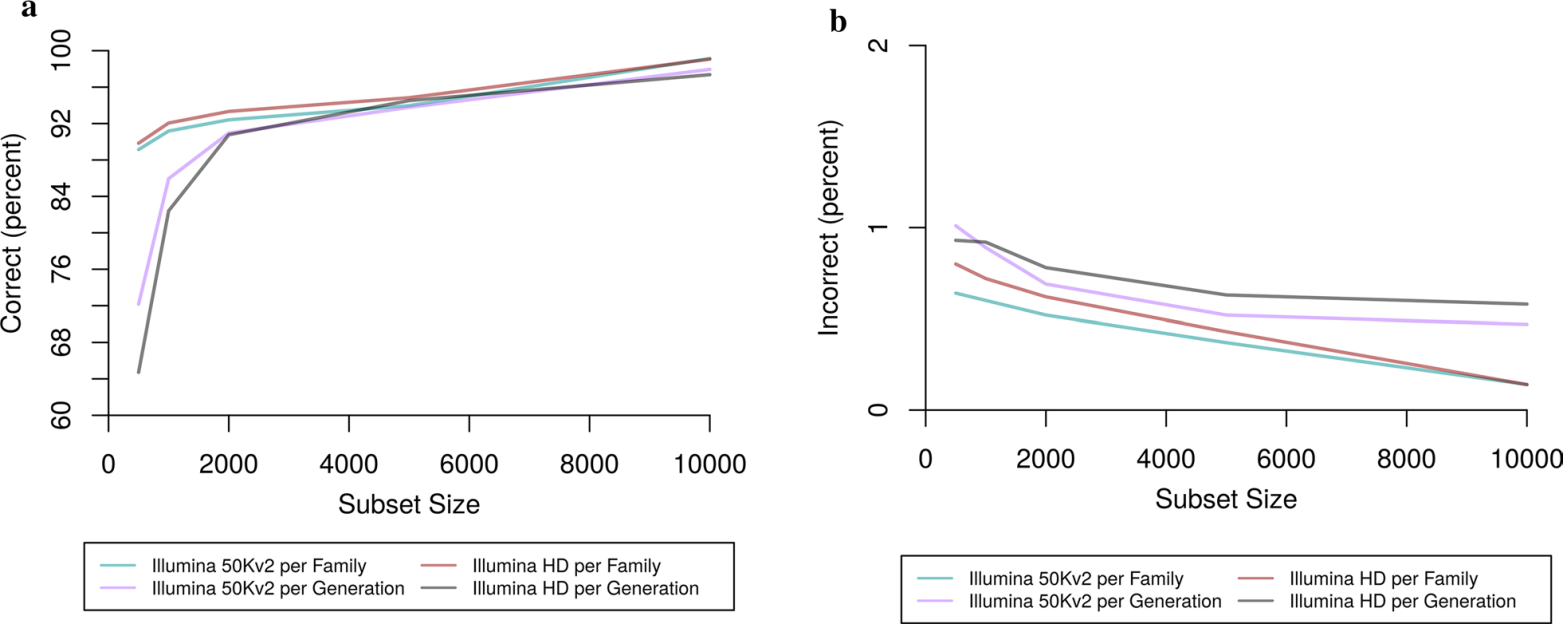
Phasing accuracy for a range of subset sizes. **a** Percentage of correctly phased alleles at heterozygous loci. **b** Percentage of incorrectly phased alleles at heterozygous loci

Subset size was expected to have a significant effect on the accuracy of phasing, as it directly influences the number of surrogates that are found. To test this, we evaluated subset sizes of between 500 and 10,000 individuals (Fig. 4) and Additional file 1: Tables S5–S8). For both the Illumina 50 Kv2 and Illumina HD scenarios, accuracy increased as the subset size increased. For the Illumina 50 Kv2 per population scenario, the percentage of correctly phased alleles at heterozygous loci increased from 89.1 to 99.2% as subset size increased from 500 to 10,000 individuals. For the Illumina HD per population scenario, it increased from 89.8% (500 individuals) to 99.1% (10,000 individuals). Results for phasing in the per generation scenarios were similar. As the subset size increased from 500 to 10,000 individuals, the percentage of correctly phased alleles increased from 72.1 to 98.1% for the Illumina 50 Kv2 scenario and from 64.7 to 97.4% for the Illumina HD scenario.

Runtime was proportional to subset size (see Additional file 1: Tables S5–S8). Memory usage also increased as subset size grows. We chose to use a subset size of 5000 for the remainder of this study as a reasonable trade-off between accuracy and runtime. With subsets of this size, the percentage of correctly phased alleles at heterozygous loci was 94.0% (per population) or 93.8% (per generation) for the Illumina 50 Kv2 scenario and 94.8% (per population) or 94.5% (per generation) for the Illumina HD scenarios. The percentage of alleles phased incorrectly was 0.4% (per population) or 0.5% (per generation) for the Illumina 50 Kv2 scenarios and 0.4% (per population) or 0.6% (per generation) for the Illumina HD scenarios.

#### Accuracy, runtime, and memory usage on different dataset sizes

**Fig. 5:**
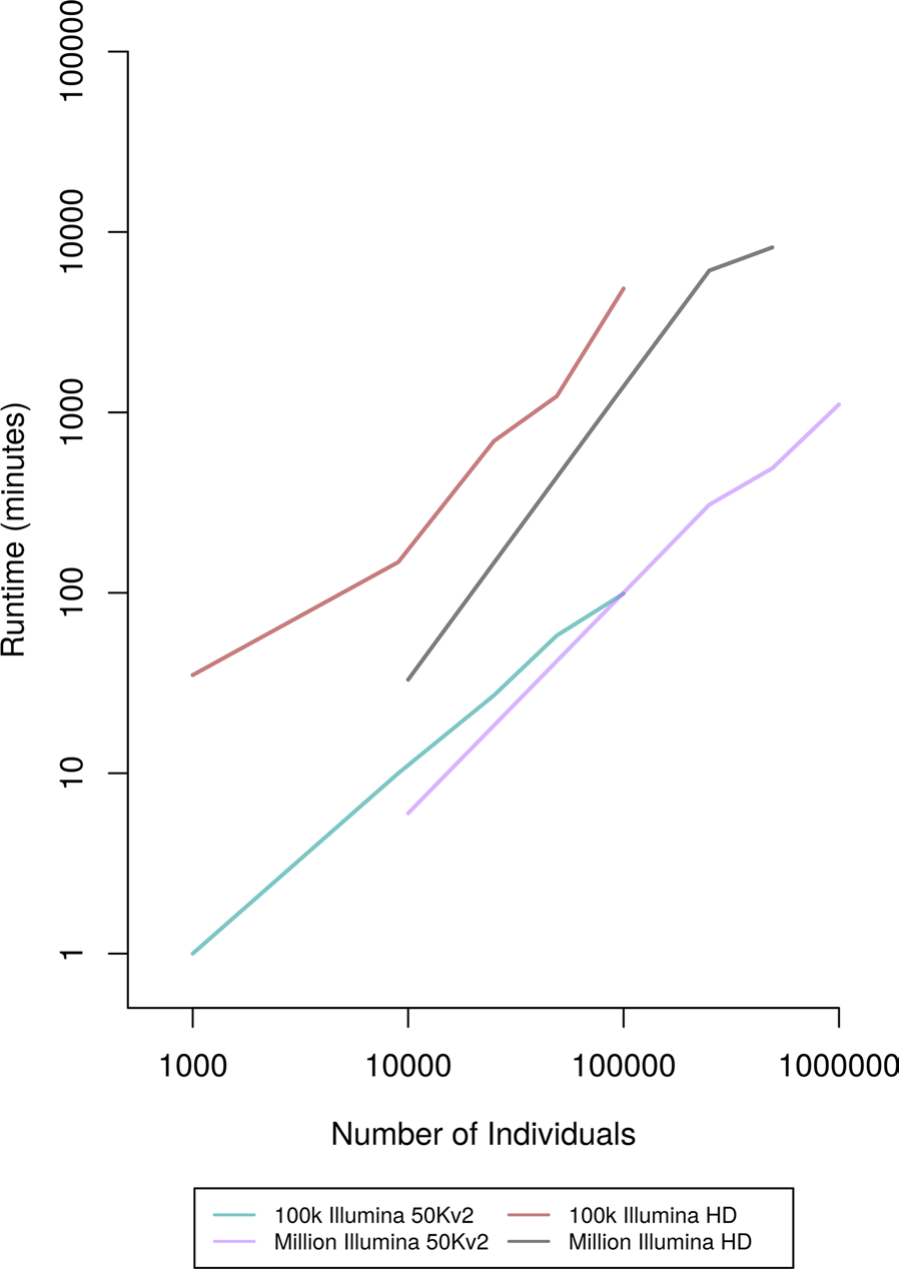
Runtime of AlphaPhase for a range of dataset sizes genotyped on two SNP arrays

**Fig. 6:**
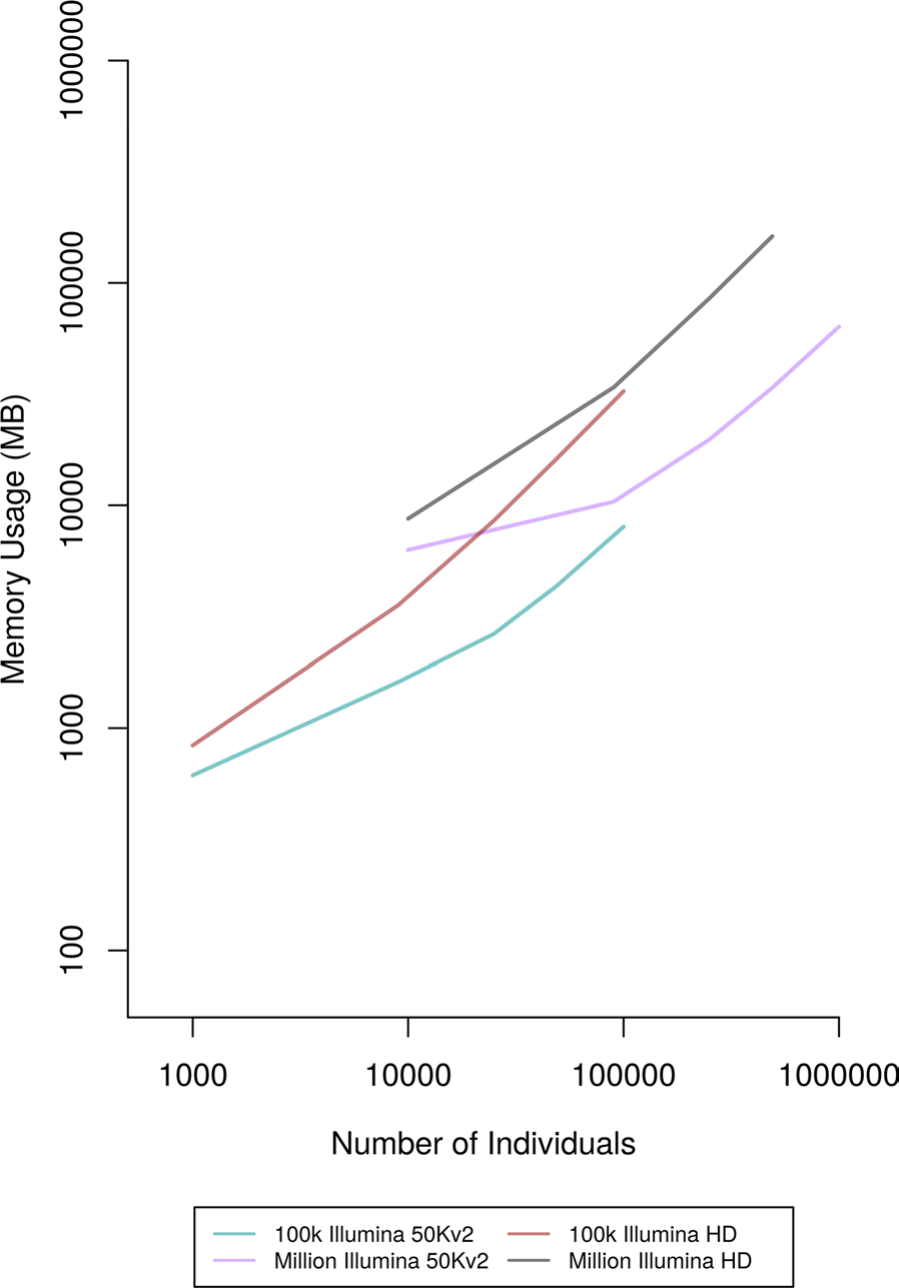
Memory usage of AlphaPhase for a range of dataset sizes genotyped on two SNP arrays

To test the performance of the improvements to the AlphaPhase LRP and HLI algorithms on datasets of different sizes, we created multiple differently sized scenarios from the 100 k and the one million datasets. Phasing accuracy was broadly comparable to the phasing accuracy observed when investigating optimal core length and subset size (see Additional file 1: Tables S9 and S10). Figure 5 shows that runtimes scaled approximately linearly with the number of individuals in a dataset. For the Illumina 50 Kv2 scenario, memory usage varied from 0.6 GB for the smallest dataset of 1000 individuals to 32 GB for a dataset of one million individuals (Fig. 6) and Additional file 1: Table S19). Comparable figures for the Illumina HD scenario were 6 GB and 325 GB (Fig. 6) and Additional file 1: Table S10).

### Heterogeneous datasets

Table 2 shows phasing accuracy, runtime and memory requirements for each of the five heterogeneous arrays per population scenarios. For the scenarios that included only MD arrays, the percentage of alleles at heterozygous loci phased correctly was between 93.2 and 95.3%, with between 1.1 and 1.5% phased incorrectly. For the two array scenarios (two-Illumina and two-mixed), runtime was under 2 h. For the three-MD scenario, runtime was under 3 h. For the two-array scenarios, memory usage was around 2.6 GB, while it was approximately 3 GB for the three-MD scenario.

**Table 2:** Phasing results from AlphaPhase for the genotype scenarios per population

Scenario	All			Heterozygous			Time (min)	Memory (MB)
	Correct	Unphased	Incorrect	Correct	Unphased	Incorrect		
Illumina 50 Kv2	98.33	1.59	0.08	93.97	5.66	0.37	47	2509
Illumina HD	98.46	1.45	0.10	94.84	4.74	0.43	119	4056
Two-Illumina	95.02	4.70	0.28	93.21	5.67	1.13	41	2448
Two-mixed	95.55	4.05	0.40	94.30	4.44	1.26	54	2552
Three-MD	96.18	3.15	0.67	95.26	3.28	1.47	118	3105
Mixed MD/HD	89.81	8.20	1.99	91.79	4.48	3.73	168	3982

We also tested a mixture of one MD array and one HD array, with nine individuals genotyped on the MD array for every individual genotyped on the HD array (mixed MD/HD). As expected, the percentage of correctly phased alleles at heterozygous loci was lower than in other scenarios, but was still 91.8%. Runtime was around 3 h and memory usage was 3.9 GB.

Table 3 shows results for the per generation scenarios, which were broadly comparable to those of the per population scenarios. Across the scenarios with only MD arrays, the percentage of correctly phased alleles at heterozygous loci was between 92.4 and 97.2%, with between 1.2 and 1.4% incorrectly phased alleles. Runtime was less than for the per population scenarios, taking under 1 h for the two-array scenarios and under 2 h for the three-MD scenarios. Memory usage was a little lower than that of the per population scenarios.

**Table 3:** Phasing results from AlphaPhase for the genotype scenarios per generation

Scenario	All			Heterozygous			Time (min)	Memory (MB)
	Correct	Unphased	Incorrect	Correct	Unphased	Incorrect		
Illumina 50 Kv2	98.10	1.78	0.12	93.78	5.69	0.52	12	1636
Illumina HD	98.40	1.46	0.14	94.52	4.85	0.63	102	3822
Two-Illumina	96.40	3.24	0.36	92.43	6.13	1.44	20	1654
Two-mixed	96.91	2.60	0.49	95.12	3.55	1.34	19	1715
Three-MD	96.78	2.34	0.87	97.23	1.54	1.23	38	1971
Mixed MD/HD	75.44	22.39	2.18	80.84	14.45	4.70	98	3863

The mixed HD/MD per generation scenario showed a lower percentage of correctly phased alleles at heterozygous loci (80.8%) compared to the per population scenario (91.8%). Runtime and memory requirements were similar.

### Comparison to Eagle2

Tables 4 and 5 show the performance of Eagle2 on per population and per generation scenarios. For all tested scenarios, Eagle2 achieved very high phasing accuracy, with the percentage of correctly phased alleles at heterozygous loci ranging from 99.25 to 99.64%, compared to from 75.44 to 98.46% for AlphaPhase. The percentage of incorrectly phased alleles varied between 0.07 and 0.75% for Eagle2 compared to between 0.37 and 4.70% for AlphaPhase. Although memory usage for Eagle 2 was similar to that for AlphaPhase, runtime was considerably longer, ranging from 709 to 2769 min for the per population scenarios, compared to from 47 to 168 min for AlphaPhase. For the per generation scenarios, Eagle2 took between 2069 and 5344 min, compared to between 12 and 102 min for AlphaPhase.

**Table 4:** Phasing results from Eagle2 for genotype scenarios per population

Scenario	All			Heterozygous			Time (min)	Memory (MB)
	Correct	Unphased	Incorrect	Correct	Unphased	Incorrect		
Illumina 50 Kv2	99.98	0.00	0.02	99.89	0.00	0.11	3325	1288
Illumina HD	99.89	0.00	0.11	99.49	0.00	0.51	7789	1495
Two-Illumina	99.98	0.00	0.02	99.89	0.00	0.11	2528	1266
Two-mixed	99.98	0.00	0.02	99.90	0.00	0.10	2339	1307
Three-MD	99.99	0.00	0.01	99.93	0.00	0.07	2069	1468
Mixed MD/HD	99.92	0.00	0.08	99.64	0.00	0.36	5344	3336

**Table 5:** Phasing results from Eagle2 for genotype scenarios per generation

Scenario	All			Heterozygous			Time (min)	Memory (MB)
	Correct	Unphased	Incorrect	Correct	Unphased	Incorrect		
Illumina 50 Kv2	99.97	0.00	0.03	99.89	0.00	0.11	709	1404
Illumina HD	99.83	0.00	0.17	99.25	0.00	0.75	2769	2639
Two-Illumina	99.97	0.00	0.03	99.88	0.00	0.12	790	1382
Two-mixed	99.98	0.00	0.02	99.89	0.00	0.11	811	1432
Three-MD	99.98	0.00	0.02	99.92	0.00	0.08	881	1585
Mixed MD/HD	99.85	0.00	0.15	99.31	0.00	0.69	1727	3573

Due to the long run times, we only tested Eagle2’s performance on different size datasets for the 100 k Illumina 50 Kv2 scenarios (see Additional file 1: Table S11). In all scenarios, Eagle2 achieved phasing accuracy at heterozygous loci of over 99.99%, compared to between 97.6 and 99.67% for AlphaPhase, but again this came at the cost of increased runtime, with the 100 k animal scenario taking 10,931 min, compared to 99 min for AlphaPhase.

## Discussion

In this paper, we introduced improvements to the LRP and HLI algorithms of AlphaPhase [2] to enable phasing of very large and heterogeneous datasets in which individuals may have been genotyped on different sets of markers. We tested the performance of the revised algorithms on a range of simulated datasets and showed that AlphaPhase can be used to accurately phase datasets that contain up to one million individuals and that have been genotyped with multiple SNP arrays. In the following, we discuss the effect of: (i) core length and (ii) subset size on phasing accuracy, computational runtime, and memory use; and (iii) the impact of these improvements on the phasing of large and heterogeneous datasets.

### Effect of core length on phasing performance

In AlphaPhase, both the LRP and HLI algorithms break the genome into smaller sections of consecutive SNPs that are called cores. Then, each of the cores is phased independently of the other cores. Core length, defined as the number of SNPs in each core, has previously been shown to have a significant effect on phasing accuracy [2]. We further investigated the impact of this parameter with the modifications made to the LRP and HLI algorithms and the use of much denser SNP arrays. As expected, we found that core length has a significant effect on phasing accuracy. Short cores resulted in similar levels of phasing accuracy but accuracy started to deteriorate notably as cores got longer. We also found that phasing accuracy is a function of the length of the core in proportion to the length of the chromosome, rather than the number of SNPs it contains, and that variation of accuracy with core length was similar for both the Illumina 50 Kv2 and Illumina HD arrays when core length was expressed as a proportion of chromosome length. This is as expected, since phasing accuracy is expected to be highly affected by the presence of recombinations within a core. The latter can be approximated by the size of a core relative to the whole chromosome, and less so by the number of SNPs in a core. The reduction in accuracy observed as the core length increased is likely due to the increased chance of a core to contain a recent recombination. This would reduce the number of surrogates and thus, reduce the information available for phasing.

Both runtime and memory usage were significantly affected by core length, with runtime being approximately proportional to the number of cores and therefore, inversely proportional to core length. Memory usage also increased as the number of cores increased, although less pronounced than for runtime. For these reasons, we recommend the use of the longest possible cores that do not result in an unacceptable drop in phasing accuracy. For the bovine arrays considered in this study, our results suggest a core length of approximately 10% of the chromosome, corresponding to the core lengths used in this study of 500 SNPs when only MD arrays are used and a core length of 5000 SNPs when HD arrays are also used.

### Effect of subset size on phasing performance

The improvements of the AlphaPhase LRP algorithm developed herein, partition a large dataset into subsets and then performs LRP on each subset. A new parameter for the LRP algorithm controls the size of these subsets and our results show that this parameter can have a significant effect on phasing accuracy. A subset size of 10,000 individuals gave both the highest percentage of correctly phased alleles and the lowest percentage of incorrectly phased ones. As the subset size decreased, the proportion of correctly phased alleles decreased and the proportion of incorrectly phased loci increased. This decrease in phasing performance was likely a result of the reduction in the number of surrogates that is expected for a smaller subset, which, in turn, leads to less information with which to accurately phase.

The increase in phasing accuracy that resulted from increasing subset size, however, had a significant cost in terms of runtime. We found an approximately linear relationship between the size of the subset and runtime. Consequently, there was a trade-off between runtime and phasing accuracy. As the size of subsets got larger, the number of incorrectly phased alleles appeared to begin to plateau for a subset size of 5000 or more, but runtime started to increase significantly. For the datasets evaluated here, the subset size for which this value occurred seemed to be largely invariant to total dataset size, and thus we suggest that a subset size of 5000 is an appropriate compromise. The optimal size for datasets with a different structure, such as in human populations, in which individuals are likely less related than in the datasets evaluated here, warrants further investigation.

Phasing of large datasets is likely computationally expensive for any phasing method due to the large number of individuals involved. Our results suggest that there is sufficient information in small subsets from a larger dataset to allow a significant number of alleles to be phased accurately. This suggests that breaking the phasing of large datasets into subsets before concatenating the results could also be beneficial for other phasing methods, such as those based on probabilistic models.

### Ability of AlphaPhase to phase large heterogeneous datasets

Our results show that running heuristic phasing on very large datasets, such as those now available for humans [12] or cattle [6, 13, 14], is feasible. AlphaPhase took less than one day and 32 GB of memory to phase one chromosome for one million animals genotyped on a simulated Illumina 50 Kv2 array. To phase one chromosome for one million animals genotyped on a simulated Illumina HD array took 23 days and 325 GB of memory. As our simulated dataset consisted of a single chromosome, total runtime for all chromosomes will be approximately 20 to 30 times greater than this. However, as AlphaPhase can phase multiple cores in parallel, the wall clock time can be reduced significantly. For the scenarios considered here, nine or ten cores were involved, so we can expect wall clock time to be roughly one-ninth or one tenth of the total processor time, provided the nine or ten processors are used in parallel.

We also compared the performance of AlphaPhase with Eagle2, a phasing method that is often used in human genetics, and found that Eagle2 had markedly higher phasing accuracy for all scenarios investigated, but at the cost of an order of magnitude longer runtime. However, Eagle2 was able to obtain lower runtimes than previous probabilistic phasing algorithms such as Shapeit2, by using the Positional Burrows Wheeler Transform [33] to create an efficient, searchable representation of the haplotype reference library.

Previous work [22] has shown that FImpute is both fast and accurate, although other studies (e.g. [25]) suggested that the algorithm with the best performance varies per dataset. As both the datasets and accuracy methods used in this paper differ from those in previous studies, it is difficult to draw any firm conclusions, although the comparisons suggest that users should consider several algorithms, including FImpute and AlphaPhase, for phasing large animal datasets quickly and accurately.

The ability to phase datasets that are genotyped using multiple different arrays is important, since datasets are increasingly likely to consist of individuals that are genotyped using different arrays due to the increase in the number of available arrays for commonly genotyped species. Previously, artificial insemination dairy sires would be genotyped on what would be now considered medium-density chips. However, more recently dairy sires are increasingly genotyped using high-density chips.

Results from analysis of the MD heterogeneous array scenarios show that the percentage of incorrectly phased alleles was generally only slightly worse for heterogeneous datasets that consist of individuals genotyped on multiple MD arrays than for homogeneous datasets, although in many scenarios the heterogeneous arrays phased slightly more alleles correctly. However, this increase in percentage of correctly phased alleles came at the cost of phasing more alleles incorrectly as well.

The extended version of AlphaPhase presented in this study can also accurately phase individuals genotyped on a mixture of MD and HD SNP arrays. The phasing of such datasets is likely to become increasingly common, as it is desirable to continue to use the data already collected using MD arrays as the use of HD arrays grows. Although the phasing accuracy for heterogeneous datasets was often lower than when individuals were genotyped on a single SNP array, the percentage of correctly phased alleles was still higher than 91% in all scenarios tested other than the mixed MD/HD per generation scenario. In this scenario, many individuals had large amounts of missing data because they were genotyped on the MD rather than HD array, and thus phasing was expected to be more difficult.

## Conclusions

We have modified the LRP and HLI algorithms to allow phasing of large heterogeneous datasets. These modifications are implemented in AlphaPhase version 1.3.7 (available from http://alphagenes.roslin.ed.ac.uk/) and allow the phasing of millions of individuals that are genotyped on multiple SNP arrays.

## Supplementary information

**Additional file 1: Table S1.** Illumina 50 Kv2 per population phasing results from AlphaPhase for a range of core lengths. **Table S2.** Illumina 50 Kv2 per generation results from AlphaPhase for a range of core lengths. **Table S3.** Illumina HD per population results from AlphaPhase for a range of core lengths. **Table S4.** Illumina HD per generation results from AlphaPhase for a range of core lengths. **Table S5.** Illumina 50 Kv2 per population results from AlphaPhase for a range of subset sizes. **Table S6.** Illumina 50 Kv2 per generation results from AlphaPhase for a range of subset sizes. **Table S7.** Illumina HD per population results from AlphaPhase for a range of subset sizes. **Table S8.** Illumina HD per generation results from AlphaPhase for a range of subset sizes. **Table S9.** Illumina 50 Kv2 results from AlphaPhase for scenarios of different sizes. **Table S10.** Illumina HD results from AlphaPhase for scenarios of different sizes. **Table S11.** Illumina 50 Kv2 results from Eage2 for scenarios of different sizes.

## Data Availability

An implementation of our algorithm, in the software package AlphaPhase, is available from the authors’ website, https://alphagenes.roslin.ed.ac.uk/wp/software-2/alphaphase/, and is free for academic use.
