# Supplementary material for: Extending long-range phasing and haplotype library imputation algorithms to large and heterogeneous datasets
Source: Genet Sel Evol. 2020 Jul 8;52:38. doi: 10.1186/s12711-020-00558-2 (PMC7346379; doi:10.1186/s12711-020-00558-2)
Supplement: Supplementary file 1 — Additional file 1: Table S1. Illumina 50 Kv2 per population phasing results from AlphaPhase for a range of core lengths. Table S2. Illumina 50 Kv2 per generation results from AlphaPhase for a range of core lengths. Table S3. Illumina HD per population results from AlphaPhase for a range of core lengths. Table S4. Illumina HD per generation results from AlphaPhase for a range of core lengths. Table S5. Illumina 50 Kv2 per population results from AlphaPhase for a range of subset sizes. Table S6. Illumina 50 Kv2 per generation results from AlphaPhase for a range of subset sizes. Table S7. Illumina HD per population results from AlphaPhase for a range of subset sizes. Table S8. Illumina HD per generation results from AlphaPhase for a range of subset sizes. Table S9. Illumina 50 Kv2 results from AlphaPhase for scenarios of different sizes. Table S10. Illumina HD results from AlphaPhase for scenarios of different sizes. Table S11. Illumina 50 Kv2 results from Eage2 for scenarios of different sizes. [file 12711_2020_558_MOESM1_ESM.docx]

**Table S1**

| **Core length** | **All loci** | | | **Heterozygous loci** | | | **Time (min)** | **Memory (MB)** |
| --- | --- | --- | --- | --- | --- | --- | --- | --- |
|  | **Correct** | **Unphased** | **Incorrect** | **Correct** | **Unphased** | **Incorrect** |  |  |
| 50 | 97.61 | 2.31 | 0.08 | 89.46 | 10.17 | 0.37 | 1726 | 6782 |
| 100 | 97.87 | 2.06 | 0.07 | 90.90 | 8.78 | 0.31 | 755 | 4485 |
| 200 | 98.09 | 1.85 | 0.06 | 92.25 | 7.47 | 0.28 | 277 | 3264 |
| 500 | 98.33 | 1.59 | 0.08 | 93.97 | 5.66 | 0.37 | 47 | 2509 |
| 1000 | 98.29 | 1.57 | 0.12 | 94.40 | 4.96 | 0.63 | 18 | 1805 |

## Table S2

| **Core length** | **All loci** | | | **Heterozygous loci** | | | **Time (min)** | **Memory (MB)** |
| --- | --- | --- | --- | --- | --- | --- | --- | --- |
|  | **Correct** | **Unphased** | **Incorrect** | **Correct** | **Unphased** | **Incorrect** |  |  |
| 50 | 98.23 | 1.49 | 0.28 | 92.27 | 6.46 | 1.27 | 80 | 3688 |
| 100 | 98.28 | 1.55 | 0.17 | 92.86 | 6.37 | 0.78 | 28 | 2483 |
| 200 | 98.02 | 1.87 | 0.10 | 92.63 | 6.90 | 0.46 | 16 | 1869 |
| 500 | 98.10 | 1.75 | 0.12 | 93.78 | 5.69 | 0.52 | 12 | 1636 |
| 1000 | 97.92 | 1.87 | 0.20 | 92.07 | 7.02 | 0.92 | 10 | 1541 |

## Table S3

| **Core length** | **All loci** | | | **Heterozygous loci** | | | **Time (min)** | **Memory (MB)** |
| --- | --- | --- | --- | --- | --- | --- | --- | --- |
|  | **Correct** | **Unphased** | **Incorrect** | **Correct** | **Unphased** | **Incorrect** |  |  |
| 500 | 98.03 | 1.90 | 0.07 | 91.49 | 8.19 | 0.33 | 1752 | 4385 |
| 1000 | 98.13 | 1.82 | 0.05 | 92.23 | 7.53 | 0.23 | 648 | 4159 |
| 2000 | 98.33 | 1.62 | 0.05 | 93.54 | 6.22 | 0.24 | 272 | 4146 |
| 5000 | 98.46 | 1.45 | 0.10 | 94.84 | 4.74 | 0.43 | 119 | 4056 |
| 10,000 | 98.13 | 1.71 | 0.16 | 93.89 | 5.42 | 0.69 | 91 | 4037 |

## Table S4

| **Core length** | **All loci** | | | **Heterozygous loci** | | | **Time (min)** | **Memory (MB)** |
| --- | --- | --- | --- | --- | --- | --- | --- | --- |
|  | **Correct** | **Unphased** | **Incorrect** | **Correct** | **Unphased** | **Incorrect** |  |  |
| 500 | 98.41 | 1.36 | 0.23 | 93.26 | 5.72 | 1.03 | 305 | 4385 |
| 1000 | 98.27 | 1.62 | 0.11 | 93.30 | 6.22 | 0.48 | 187 | 3928 |
| 2000 | 98.09 | 1.83 | 0.08 | 93.46 | 6.19 | 0.35 | 136 | 3852 |
| 5000 | 98.40 | 1.46 | 0.14 | 94.52 | 4.85 | 0.63 | 102 | 3922 |
| 10,000 | 96.90 | 2.89 | 0.21 | 86.82 | 12.25 | 0.93 | 82 | 3941 |

## Table S5

| **Subset size** | **All loci** | | | **Heterozygous loci** | | | **Time (min)** | **Memory (MB)** |
| --- | --- | --- | --- | --- | --- | --- | --- | --- |
|  | **Correct** | **Unphased** | **Incorrect** | **Correct** | **Unphased** | **Incorrect** |  |  |
| 500 | 97.02 | 2.83 | 0.14 | 89.14 | 10.21 | 0.64 | 4 | 1246 |
| 1000 | 97.56 | 2.30 | 0.13 | 91.18 | 8.22 | 0.60 | 6 | 1255 |
| 2000 | 97.90 | 1.98 | 0.12 | 92.41 | 7.08 | 0.52 | 14 | 1424 |
| 5000 | 98.33 | 1.59 | 0.08 | 93.97 | 5.66 | 0.37 | 47 | 2509 |
| 10,000 | 99.81 | 0.16 | 0.03 | 99.15 | 0.71 | 0.14 | 53 | 3525 |

## Table S6

| **Subset size** | **All loci** | | | **Heterozygous loci** | | | **Time (min)** | **Memory (MB)** |
| --- | --- | --- | --- | --- | --- | --- | --- | --- |
|  | **Correct** | **Unphased** | **Incorrect** | **Correct** | **Unphased** | **Incorrect** |  |  |
| 500 | 93.42 | 6.35 | 0.22 | 72.16 | 26.83 | 1.01 | 2 | 1276 |
| 1000 | 96.42 | 3.39 | 0.20 | 85.95 | 13.16 | 0.89 | 4 | 1294 |
| 2000 | 97.40 | 2.44 | 0.15 | 90.96 | 8.35 | 0.69 | 5 | 1302 |
| 5000 | 98.10 | 1.78 | 0.12 | 93.78 | 5.69 | 0.52 | 12 | 1636 |
| 10,000 | 99.54 | 0.35 | 0.10 | 97.95 | 1.58 | 0.47 | 24 | 2970 |

## Table S7

| **Subset size** | **All loci** | | | **Heterozygous loci** | | | **Time (min)** | **Memory (MB)** |
| --- | --- | --- | --- | --- | --- | --- | --- | --- |
|  | **Correct** | **Unphased** | **Incorrect** | **Correct** | **Unphased** | **Incorrect** |  |  |
| 500 | 97.20 | 2.62 | 0.18 | 89.84 | 9.36 | 0.80 | 45 | 3723 |
| 1000 | 97.73 | 2.10 | 0.16 | 92.06 | 7.22 | 0.72 | 51 | 3724 |
| 2000 | 98.06 | 1.80 | 0.14 | 93.33 | 6.04 | 0.62 | 72 | 3723 |
| 5000 | 98.46 | 1.45 | 0.10 | 94.84 | 4.74 | 0.43 | 119 | 4056 |
| 10,000 | 99.79 | 0.18 | 0.03 | 99.07 | 0.79 | 0.14 | 230 | 4762 |

## Table S8

| **Subset size** | **All loci** | | | **Heterozygous loci** | | | **Time (min)** | **Memory (MB)** |
| --- | --- | --- | --- | --- | --- | --- | --- | --- |
|  | **Correct** | **Unphased** | **Incorrect** | **Correct** | **Unphased** | **Incorrect** |  |  |
| 500 | 91.83 | 7.97 | 0.21 | 64.72 | 34.35 | 0.93 | 38 | 3723 |
| 1000 | 95.76 | 4.03 | 0.20 | 82.41 | 16.66 | 0.92 | 42 | 3724 |
| 2000 | 97.56 | 2.26 | 0.17 | 90.77 | 8.44 | 0.78 | 55 | 3724 |
| 5000 | 98.40 | 1.46 | 0.14 | 94.52 | 4.85 | 0.63 | 102 | 3822 |
| 10,000 | 99.42 | 0.46 | 0.13 | 97.37 | 2.05 | 0.58 | 185 | 4741 |

## Table S9

| **Dataset** | **Families**$\times$**generations** | **Number of individuals** | **All loci** | | | **Heterozygous loci** | | | **Time (min)** | **Memory (MB)** |
| --- | --- | --- | --- | --- | --- | --- | --- | --- | --- | --- |
|  |  |  | **Correct** | **Unphased** | **Incorrect** | **Correct** | **Unphased** | **Incorrect** |  |  |
| 100k | 1×1 | 1000 | 99.67 | 0.25 | 0.08 | 98.57 | 1.08 | 0.35 | 1 | 613 |
| 100k | 3×3 | 9000 | 97.77 | 2.17 | 0.06 | 92.53 | 7.20 | 0.27 | 10 | 1611 |
| 100k | 5×5 | 25,000 | 97.60 | 2.30 | 0.10 | 91.70 | 7.88 | 0.42 | 27 | 2641 |
| 100k | 7×7 | 49,000 | 97.73 | 2.13 | 0.13 | 91.80 | 7.62 | 0.58 | 58 | 4343 |
| 100k | 10×10 | 100,000 | 97.62 | 2.22 | 0.16 | 90.98 | 8.32 | 0.70 | 99 | 8014 |
| One million | 1×1 | 10,000 | 98.68 | 1.25 | 0.07 | 95.60 | 4.11 | 0.29 | 6 | 836 |
| One million | 3×3 | 90,000 | 97.01 | 2.91 | 0.08 | 89.92 | 9.74 | 0.34 | 88 | 3557 |
| One million | 5×5 | 250,000 | 97.50 | 2.40 | 0.10 | 91.06 | 8.48 | 0.46 | 308 | 8531 |
| One million | 7×7 | 490,000 | 97.66 | 2.21 | 0.13 | 91.17 | 8.24 | 0.59 | 490 | 16,227 |
| One million | 10×10 | 1,000,000 | 97.57 | 2.26 | 0.17 | 90.18 | 9.03 | 0.79 | 1107 | 32,576 |

## Table S10

| **Dataset** | **Families**$\times$**generations** | **Number of individuals** | **All loci** | | | **Heterozygous loci** | | | **Time (min)** | **Memory (MB)** |
| --- | --- | --- | --- | --- | --- | --- | --- | --- | --- | --- |
|  |  |  | **Correct** | **Unphased** | **Incorrect** | **Correct** | **Unphased** | **Incorrect** |  |  |
| 100k | 1×1 | 1000 | 99.53 | 0.35 | 0.12 | 97.99 | 1.51 | 0.50 | 35 | 6299 |
| 100k | 3×3 | 9000 | 97.89 | 2.03 | 0.07 | 93.71 | 5.97 | 0.32 | 148 | 10,381 |
| 100k | 5×5 | 25,000 | 98.11 | 1.78 | 0.11 | 93.62 | 5.89 | 0.50 | 693 | 19,747 |
| 100k | 7×7 | 49,000 | 98.07 | 1.78 | 0.15 | 92.97 | 6.34 | 0.68 | 1231 | 33,771 |
| 100k | 10×10 | 100,000 | 97.79 | 2.02 | 0.19 | 91.26 | 7.87 | 0.87 | 4860 | 63,574 |
| One million | 1×1 | 10,000 | 98.83 | 1.10 | 0.07 | 96.47 | 3.22 | 0.31 | 118 | 8704 |
| One million | 3×3 | 90,000 | 97.19 | 2.71 | 0.09 | 91.32 | 8.28 | 0.40 | 793 | 33,929 |
| One million | 5×5 | 250,000 | 98.01 | 1.86 | 0.12 | 93.03 | 6.41 | 0.55 | 3687 | 85,226 |
| One million | 7×7 | 490,000 | 97.95 | 1.89 | 0.16 | 92.16 | 7.10 | 0.75 | 7026 | 162,012 |
| One million | 10×10 | 1,000,000 | 97.56 | 2.23 | 0.21 | 89.93 | 9.09 | 0.98 | 34,156 | 325,310 |

## Table S11

| **Dataset** | **Families**$\times$**generations** | **Number of individuals** | **All loci** | | | **Heterozygous loci** | | | **Time (min)** | **Memory (MB)** |
| --- | --- | --- | --- | --- | --- | --- | --- | --- | --- | --- |
|  |  |  | **Correct** | **Unphased** | **Incorrect** | **Correct** | **Unphased** | **Incorrect** |  |  |
| 100k | 1×1 | 1000 | 99.98 | 0.00 | 0.02 | 99.93 | 0.00 | 0.07 | 33 | 162 |
| 100k | 3×3 | 9000 | 99.99 | 0.00 | 0.01 | 99.94 | 0.00 | 0.06 | 1177 | 1198 |
| 100k | 5×5 | 25,000 | 99.98 | 0.00 | 0.02 | 99.93 | 0.00 | 0.07 | 6115 | 3214 |
| 100k | 7×7 | 49,000 | 99.98 | 0.00 | 0.02 | 99.91 | 0.00 | 0.09 | 8214 | 3277 |
| 100k | 10×10 | 100,000 | 99.98 | 0.00 | 0.02 | 99.89 | 0.00 | 0.02 | 10,932 | 4144 |
